# Factors Limiting the Appropriate Use of Rabies Post-exposure Prophylaxis by Health Professionals in Brazil

**DOI:** 10.3389/fvets.2022.846994

**Published:** 2022-05-06

**Authors:** Ramiro M. da Silva, Jane Megid, Katie Hampson, Aline Alves Scarpellini Campos, Cintia S. Higashi, Fabíola S. Medeiros, Alexandra S. Pereira, Julio A. Benavides

**Affiliations:** ^1^Department of Veterinary Hygiene and Public Health, São Paulo State University, Botucatu, Brazil; ^2^Institute of Biodiversity, Animal Health and Comparative Medicine, College of Medical, Veterinary and Life Sciences, University of Glasgow, Glasgow, United Kingdom; ^3^Programa Estadual de Controle e Profilaxia da Raiva, State Department of Health of Rio Grande do Sul, Porto Alegre, Brazil; ^4^Coordenação de Promoção à Saúde, State Department of Health of Rio Grande do Norte, Natal, Brazil; ^5^Diretoria de Vigilância Epidemiológica, State Department of Health of Santa Catarina, Florianópolis, Brazil; ^6^Doctorado en Medicina de la Conservación y Centro de Investigación para la Sustentabilidad, Facultad de Ciencias de la Vida, Universidad Andres Bello, Santiago, Chile; ^7^MIVEGEC, IRD, CNRS, Université de Montpellier, Montpellier, France

**Keywords:** dogs, One Health, surveillance, questionnaires, Latin America, bites, PEP

## Abstract

Post-exposure prophylaxis (PEP) is necessary to prevent the fatal onset of rabies but requires optimization to avoid overuse in populations at risk of rabies. In Brazil, the incidence of dog bites remains high, with almost half of dog-bite patients not receiving the PEP recommended by the Ministry of Health guidelines between 2008 and 2017. In this study, we aimed to identify the factors that limit the appropriate prescribing of PEP by interviewing health professionals responsible for PEP administration and completion of the ‘Information System on Diseases of Compulsory Declaration’ (SINAN) form reporting human anti-rabies care for patients seeking health care after a dog bite. We conducted 147 questionnaires (45 questions each) in three Brazilian states (i.e., Rio Grande do Sul, Santa Catarina, Rio Grande do Norte) including questions related to the criteria used by professionals to classify a dog as “suspect” or “rabid”, knowledge on PEP prescription guidelines, SINAN and communication with veterinarians. Our analyses showed that most health professionals delivering PEP in these three states struggle to identify a rabies “suspect” dog according to the Ministry of Health guidelines, and to indicate the adequate PEP regimen, with only 11% of professionals prescribing the appropriate PEP under various dog-bite patient scenarios. PEP knowledge score was higher among professionals trained on PEP guidelines and working in facilities with the highest incidence of dog bites. In contrast, PEP knowledge scores did not vary significantly between states, and were not correlated to the professional's level of experience, the number of colleagues available at the health unit or the professional's confidence on prescribing appropriate PEP. Our results suggest that knowledge gaps in PEP administration among health professionals of Brazil can be reduced by implementing training programs to differentiate among rabies risk scenarios, prescribe the corresponding appropriate PEP and improve communication between health and veterinary authorities.

## Introduction

Rabies is a zoonotic virus that causes a progressive neurological infection with a case-fatality rate of almost 100% (1). Although deaths are preventable through the administration of timely post-exposure prophylaxis (PEP), there are no effective therapies once the virus reaches the central nervous system (2). Rabies virus has many domestic and wild reservoirs including dogs, bats, foxes, skunks and primates (3). Domestic dogs are responsible for 99% of human cases worldwide, spreading to people and other animals via mucosal contact during bites or contact between saliva and scratches (1, 4). Successful dog anti-rabies vaccination has reduced canine rabies transmission to only a few endemic areas in Latin America since 2010 (5, 6). Post- exposure prophylaxis is necessary to prevent the fatal onset of rabies in humans and must be provided if there is any suspicion of infection in the biting animal. However, PEP is costly and often has limited availability in low and middle-income countries, requiring efforts to prevent overuse of this scarce resource in at-risk populations (7).

In Latin America, despite the recent substantial progress in reducing canine rabies (6), the use and cost of PEP in patients bitten by dogs remains high. The high cost of PEP challenges the sustainability of rabies elimination programs in countries like Brazil, where more than half a million bitten patients are still treated each year (7, 8). In the last decades, Brazil achieved great progress in reducing human cases of dog-mediated rabies through vaccination campaigns in dogs and provisioning of PEP (5, 8). However, the country still spends dozens of millions of dollars annually in rabies control and prophylaxis [e.g., more than USD 35 million in 2013 (9)], although this cost has not been accurately estimated in the country. Without reducing PEP efficiency, a previous study suggested that PEP costs could be reduced by at least USD 6 million if the Brazilian Ministry of Health guidelines and WHO's 2018 new recommendations were followed (8). The reasons why health professionals still provide inappropriate PEP in around half of bite patients remain unknown (8).

Brazil uses a national standard surveillance protocol to report patients bitten or injured by any mammal including dogs since 1998, known as the ‘Human Anti-rabies care’ section of the “Information System on Diseases of Compulsory Declaration” (SINAN) form. The SINAN form includes 60 fields with information on the epidemiological background of the animal and the PEP protocol applied. The epidemiological background contains information to guide the correct PEP administration including the type of exposure, characteristics of the injury (e.g., single or multiple wounds, location in the body and severity), the health condition of the dog assessed by the health professional after interviewing the patient (e.g., healthy, rabid, suspect for rabies or dead/disappeared), whether it is possible to observe the dog over a 10-day period, the number of doses of vaccine applied, and if Rabies Immune Globulin (RIG) was administered (8).

The health condition of the dog and characteristics of the wound are essential criteria to guide the indication of PEP (10). Analysis of SINAN forms have previously shown that the incidence of dog bites in Brazil remained relatively constant in recent years, although reports of dogs classified as “suspect” for rabies increased from 17% in 2008 to 25% in 2017 (8). Despite most bites being caused by healthy dogs, almost half of the patients received inappropriate PEP (defined here as not following the PEP recommended by the Ministry of Health and state guidelines), which could be driven by over-assessing a healthy dog as “suspect” for rabies (8, 11). For example, health professionals often report suspect cases of canine rabies in states where dog-mediated rabies is thought to be absent, resulting in more than 2,000 “false-positives” cases reported in SINAN between 2008-2017 compared to the official Regional Information System for the Epidemiological Surveillance of Rabies (SIRVERA) surveillance system, where data is previously curated at the national level before submission (8). Inadequate completion of the SINAN form may either mis-identify cases that pose a real risk (e.g., classifying a dog acquiring rabies from a bat as a “healthy” dog) resulting in not administering PEP when needed, or increase healthcare costs when PEP is unnecessary applied.

Primary healthcare staff that are the first to interact with dog-bite victims play a crucial role in the prevention of rabies (12). Surveys have been widely used in public health to quantify rabies knowledge by health professionals and identify drivers that affect PEP administration (12–14). For example, surveys have shown that only 39% of surveyed physicians in the United States could identify a correct PEP schedule (13) and highlighted poor awareness among nurses on the potential risk of rabies from licks and scratches in India (12). However, to date, no study has evaluated rabies knowledge of health professionals in Brazil and the factors that could hinder rabies prevention measures following dog bites.

In this study, we focused on understanding PEP knowledge and administration by health professionals in three Brazilian states where canine rabies is not currently reported, including the southern states of Rio Grande do Sul (RS) and Santa Catarina (SC), and the northeast state of Rio Grande do Norte (RN). The states of RS and SC have been self-declared as controlled for the canine-rabies variant (AgV2) since 1988 and 1996 respectively (15, 16). Despite these two states not having reported a case of rabies from the canine variant over the last decade, rabies cases in dogs transmitted by bats have been reported (i.e., 2007 in RS and 2016 in SC). RN reported its last case of the canine-rabies variant in dogs in 2016 (17). Additionally, 11 cases in dogs were reported between 2011 and 2016, and 6 cases of the rabies canine variant (AgV2) were detected in the wild crab-eating fox *Cerdocyon thous* between 2015 and 2020 (17, 18). Other wildlife reservoirs of rabies (e.g., bats, the common marmoset *Callithrix jacchus* or foxes) are present in all three states (16–19). We aimed to determine (i) the criteria used by health professionals to identify a dog as rabid or suspicious for rabies, (ii) the factors correlated with prescribing appropriate PEP by health professionals, and (iii) describe how the SINAN form is completed by health professionals.

## Methods

### Data Collection

Between March 2019 and April 2020, we conducted questionnaires in Portuguese among health professionals responsible for filling the SINAN form and prescribing PEP including doctors, nurses and technicians in public health centers of the states of RS, SC and RN. In each state, we first obtained a list of all public health centers in each municipality administering PEP and completing the SINAN form from the state's health secretary. Each health center was then ranked according to the total number of patients/notifications received in 2017, and we then selected up to 10 municipalities with the most notifications (i.e., “high notification center group”) and up to 25 municipalities with the fewest (i.e., “low notification center group” with 1–4 notifications/year). We aimed to undertake ~25 questionnaires per group per state, which was assessed by the state's health secretaries as an attainable objective during a 1 month-collection period in each state. In municipalities including several health centers, we invited health centers with the highest notification rates to participate, not exciding 5 health centers per municipality. All professionals willing to participate in the study were interviewed at each health center. We interviewed all available professionals until the 1-month period was completed. Data were entered using the free open source KoBoToolbox software (https://www.kobotoolbox.org/) on a mobile phone. All participants were given a document describing the study objectives and a data confidentiality statement before confirming their voluntary enrolment in the study. Professionals were given the team's contact information for further inquiries.

Our questionnaire included 45 questions divided into 4 sections. Section 1 included 13 questions on the criteria used to assess the dog's health condition (6 questions), the 10-day observation period (4 questions), bite severity (1 question) and the professional's trust in correctly assessing the dog's health condition (2 questions). Section 2 included 12 questions on PEP administration, with four questions related to specific simulated scenarios of dog bites where the professional was asked to choose the appropriate PEP according to the Ministry of Health guidelines (20). Section 3 included 13 questions on how and when the SINAN form was completed (9 questions), the utility/feedback of the SINAN results (2 questions), and 2 questions related to PEP training. Section 4 included 7 questions regarding the current dog rabies and PEP situation in the area. Most questions were multiple-choice answers and if the answer given by the participant was not included in the available choices, it was recorded in a “other response” category along with the specific answer. All questions are detailed in Supplementary Table S1. The questionnaire was first validated on five health professionals from RS contacted by the state's health secretary to test its clarity and comprehension, and revised where necessary. To maintain the professional's anonymity, we did not record individual variables during questionnaires (e.g., age, gender or specific profession).

### Data Analyses

We built a PEP Knowledge Score (KS) using four questions aiming to assess whether the health professional could identify the appropriate PEP according to the Ministry of Health and state guidelines under four different bite patient scenarios, which were discussed with professionals from the health secretary of RS. The PEP KS ranged from 0 (four incorrect answers) to 4 (four correct answers). The four questions related to hypothetical scenarios are given in Supplementary Table S1 (questions 15–18). We then tested the correlation between the PEP KS and several variables obtained during the questionnaires including the state ID, whether the professional was in a high or low notification health center, the number of years working in that center, if he/she had received PEP training, the number of colleagues at the center that can complete the SINAN form or administer PEP, and the professional's level of confidence in PEP decisions.

We performed a multivariable analysis to test the influence of the above variables on the PEP KS using a generalized linear model (GLM) with Poisson errors, given the count nature of our response variable (i.e., KS from 0 to 4). All statistical analyses were conducted in R 3.4.3 (21). The GLM model was build using the *glm* function in R. Variable significance was tested using the Wald's test.

### Ethical Approval

The study was approved by the Ethics Committee in Research of the Faculty of Medicine of Botucatu at the São Paulo State University (UNESP), representing the National Platform Brazil of the National Committee of Health of Brazil (project number: 3.029.348).

## Results

A total of 147 questionnaires were conducted, including 80 questionnaires conducted in high notification centers from 17 cities and 67 questionnaires in low notification centers from 46 cities. 62 questionnaires were performed in RS, 51 in SC and 34 in RN. Questionnaires were conducted through an individual interview performed in person at a health facility (*N* = 69) or by phone (*N* = 78).

### Evaluation of the Dog's Health Condition and 10-Day Observation Period

**Figure 1:**
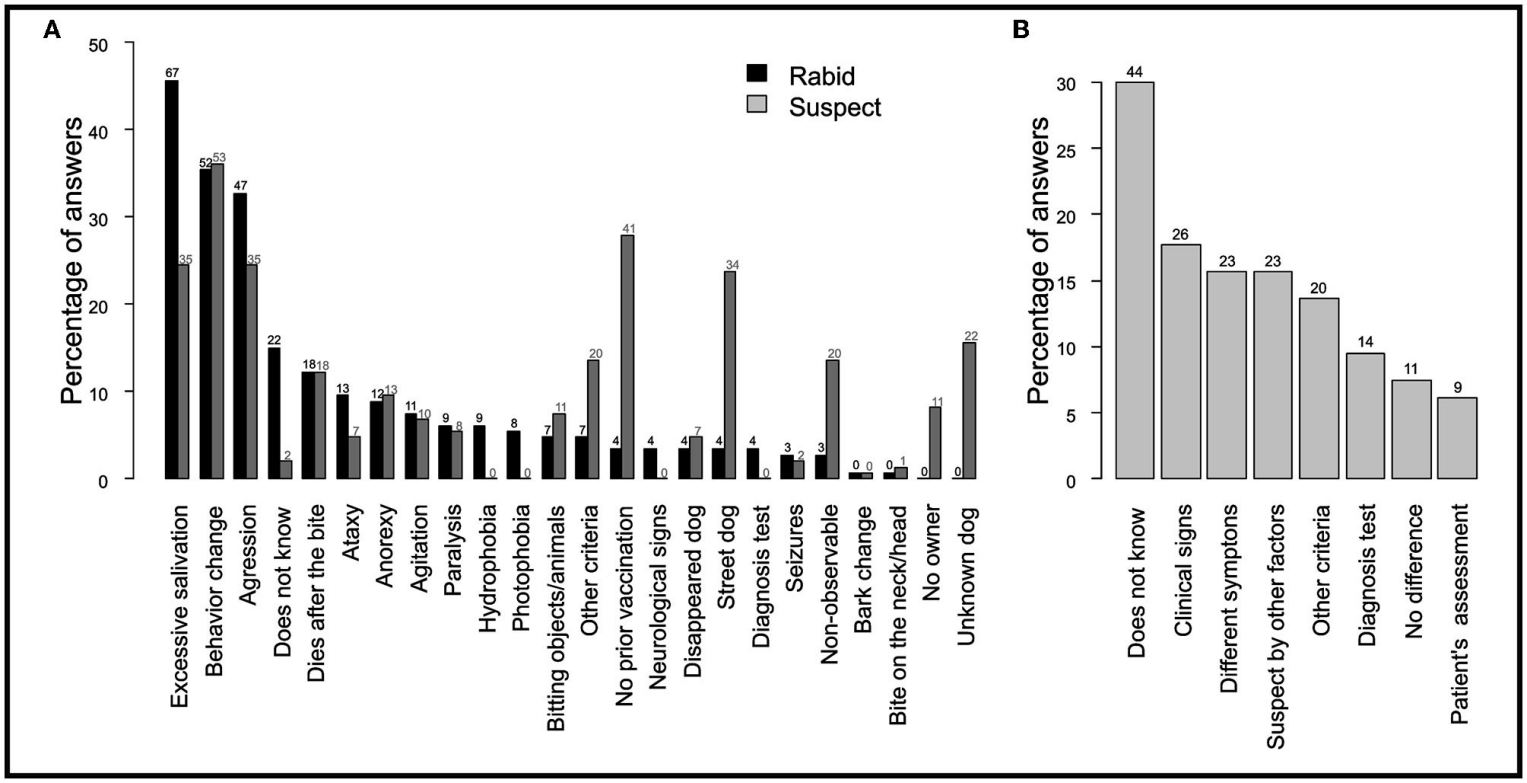
Criteria used by health professionals to classify a dog as “suspect” of rabies or “rabid.” Reported criteria used by health professionals to **(A)** define a “rabid” and “suspect” dog, and **(B)** distinguish between a “suspect” and “rabid” dog. The y-axis represents the percentage of professionals that included that criterion (out of 147 respondents). Respondents could mention more than one criteria.

Professionals used 24 different criteria to classify a dog as either “rabid” or “suspect” in the SINAN form. The percentage of each answer per criterion are provided in Figure 1A. Common distinctive signs of a rabid dog such as excessive salivation (46%, *N* = 67 out of 147 respondents), behavioral change (35%, *N* = 52) and aggression (33%, *N* = 48) were among the most used criteria to define a dog as “rabid” by health professionals. In contrast, 15% (*N* = 22) did not know a specific criterion to classify a dog as rabid. Similar criteria were used to assess a dog as “suspect,” except for four criteria that were more used to classify a dog as “suspect” including “no prior vaccination” (28%, *N* = 41), being a “street dog” (24%, *N* = 35), “a non-observable dog” (14%, *N* = 20) or an “unknown” dog (16%, *N* = 23). When asked how often they could reliably assess a dog's health condition, 49% (*N* = 172) affirmed to often know how to assess it, 26% (*N* = 38) only occasionally and 7% (*N* = 10) never. Eight different answers were provided when professionals were asked how to differentiate between a “rabid” and a “suspect of rabies” dog. The percentage of answers per response are provided in Figure 1B. While 30% (*N* = 44) of professionals indicated that they did not know how to differentiate a “suspect” from a “rabid” dog, only 18% (*N* = 26) used ‘specific clinical signs’, 10% (*N* = 14) differentiated both categories based on laboratory testing, and 7% (*N* = 11) declared that there was no difference between the categories.

**Figure 2:**
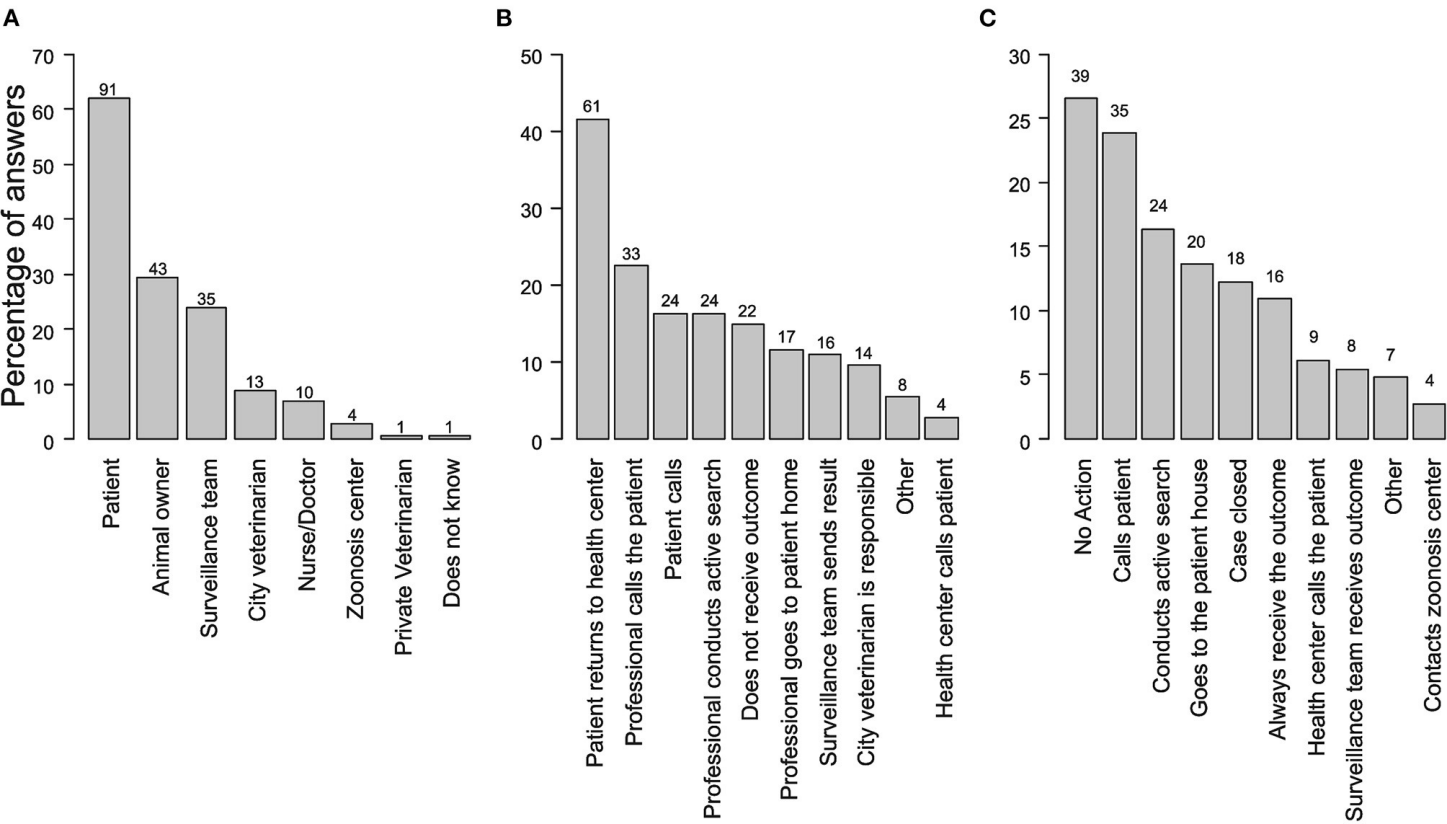
Actions of health professionals related to a dog's 10-day period observation required to assess its rabies status. **(A)** Person in charge of the observation period. Respondents could mention more than one criterion. **(B)** Reported actions allowing the health professional to receive the outcome of the 10-day observation period **(C)** Action taken by the health professional in the case that the observation outcome is not received.

The majority (62%, *N* = 91) of professionals reported that the patient is responsible for the 10-day period of observation of the dog (Figure 2A). The observation outcome was primarily communicated (41%, *N* = 61) when a patient returned to the health unit, while 15% (*N* = 22) of health professionals did not receive feedback on the observation (Figure 2B). Not acting (27%, *N* = 39) and calling the patient (24%, *N* = 35) were the most common actions if a professional did not receive the observation outcome (Figure 2C).

### Indication of PEP

Health professional's Knowledge Score (KS) on the correct application of PEP to four different scenarios ranged from 0 to 4, with 11% obtaining a KS = 0, 23% a KS = 1, 31% a KS = 2, 24% a KS = 3 and 11% a KS = 4. No overdispersion was found on the GLM model studying the influence of several variables on KS (Dispersion test, *AER* library in R, *p* > 0.05). We present the full model including all variables (AIC = 463). The reduced model including only significant variables (*p* < 0.05) provided similar estimates and AIC (459) than the full model. The KS was lower in low notification cities (GLM, estimate = −0.31, *p* = 0.01), and increased among health professionals who received specific PEP training (GLM, estimate = 0.30, *p* = 0.02) (Table 1). In contrast, the confidence level of the professional on prescribing PEP [tested as a binominal variable (none/confidence) or a multimodal variable (none, low, medium or high confidence)], the number of years completing the SINAN form, the number of colleagues completing the form/administering PEP at the center and the center's state did not significantly correlate with the KS (GLM, *p* > 0.05) (Table 1).

**Table 1:** Factors influencing the professional's PEP Knowledge Score (KS).

**Variables**	**Estimate**	**Standard Error**	***P*-value**
Training on PEP administration (yes)	0.30	0.13	0.02^*^
Rio Grande do Sul	−0.22	0.16	0.16
Santa Catarina	−0.20	0.17	0.23
Cities with low notification level	−0.31	0.13	0.01^*^
Number of professionals filling the SINAN form	−0.00	0.01	0.99
Number of years completing the SINAN form	−0.01	0.01	0.22
Professional's confidence on applying PEP (none)	−0.22	0.15	0.14

*Results from a generalized linear model (with Poisson distribution) testing the correlation between professional's PEP KS (0–4) and several variables. The state ID variable used the state of Rio Grande do Norte as the baseline. Professional's confidence on applying PEP was included as a binomial variable divided between “none” or “low/medium/high,” which was selected over a variable with 4 categories (i.e., none, low, medium and high) based on the model's higher AIC when including the latest*.

* *Significant effect*.

When asked to identify seven different potential wounds as “light” or “severe” according to the Ministry of Health guidelines, only 3% (*N* = 5) of professionals classified all seven wounds correctly, on which “punctiform injury without bleeding” and “multiple light wounds on the back” presented more than 60% of errors. A patient not returning to the center was the main obstacle to successfully administering the complete PEP regimen for 39% (*N* = 57) of professionals, followed by vaccine shortages (18%, *N* = 27) and a reduced number of centers administering PEP for the entire population (14%, *N* = 20).

The majority (58%, *N* = 86) of health professionals felt ‘medium confidence’ on indicating the appropriate PEP, followed by ‘low confidence’ (24%, *N* = 35) and ‘high confidence’ (16%, *N* = 23), while only 2% (*N* = 3) stated that they never felt confident. Most professionals (82%, *N* = 120) responded that they “often” or “always” knew the correct PEP to apply, 65% (*N* = 96) stated that they would call a colleague if they had a doubt, while 22% (*N* = 53) would search the available guidelines. Only 54% (*N* = 79) of professionals received training in PEP administration.

### Completion of the SINAN Form

The majority (81%, *N* = 119) of professionals reported to understand all the form fields and 71% (*N* = 105) declared to fill the entire SINAN form. Most professionals (44%, *N* = 64) took 11 to 20 min to fill out the form, followed by 0 to 10 min (38%, *N* = 56), and 21 to 60 min (18%, *N* = 27). The majority (82%, *N* = 120) did not regularly ask for help to a colleague when assessing a patient and completing the SINAN form. When asked about the multiple methods to fill up the form that they would use, the majority (62%, *N* = 91) preferred to complete the form on a computer, followed by on paper (46%, *N* = 67) or via a mobile app (22%, *N* = 32). Even though 75% (*N* = 110) of professionals did not receive feedback on the forms they sent, the majority (88%, *N* = 129) believed that the form was used to optimize PEP administration.

### One Health Interactions With Veterinarians and Knowledge of the Rabies Local Situation

The majority (66%, *N* = 97) of health professionals reported not requesting help from a veterinarian, 33% (*N* = 421) requested help to assess the 10-day period of observation, and only 8% (*N* = 11) to determine the dog's health condition. When asked how to improve interactions with veterinarians, 45% (*N* = 66) mentioned having a veterinarian in the state or city's epidemiological surveillance team could help, 10% (*N* = 14) mentioned training together with veterinarians, and 7% (*N* = 10) mentioned having more meetings with the epidemiological surveillance team. Regarding tracking secondary bites, most professionals (77%, *N* = 113) did not seek other people bitten by the same animal, 38% (*N* = 56) reported they would call the health secretary if they suspected a rabid dog, and 29% (*N* = 42) said they would not warn any authority and would just complete the form and provide PEP. A large proportion (41%, *N* = 60) of professionals did not know if dog/cat rabies cases had been reported in their state during the last 2 years, whereas 29% (*N* = 43) did not know if dog/cat rabies cases had been reported in their municipality during the last 5 years.

## Discussion

In Brazil, only half of dog-bite patients received appropriate PEP following a dog bite during the last decade (8). Our analyses showed that most health professionals delivering PEP in the states of Rio Grande do Norte, Rio Grande do Sul and Santa Catarina struggled to differentiate a “rabid” from a “suspect” dog according to the Ministry of Health guidelines and to indicate the appropriate PEP regimen, with only 11% indicating the appropriate PEP when given four different bite patient scenarios. Professionals' PEP Knowledge Score (KS) was higher in cities with higher incidence of dog bites and among professionals who were specifically trained on PEP guidelines. In contrast, PEP KS did not vary significantly between states, with the professional's experience level, the number of their colleagues at the health center or with their level of confidence on delivering appropriate PEP. Therefore, the inability to identify a rabid dog and poor PEP knowledge resulting from insufficient training of health professionals in areas of low or self-declared controlled dog-mediated rabies could be a main factor explaining the high levels of PEP misuse in Brazil.

The percentage of reported suspect dogs among dog-bite patients attending public health centers increased from 17% in 2008 to 25% in 2017 in Brazil, despite an average of just 1 to 3 laboratory-confirmed dog-mediated human rabies cases each year since 2006 (5, 8). Optimizing PEP use thus requires understanding what generates this relatively high percentage of dogs assessed as suspect for rabies. To assess the health condition of a dog and administer the appropriate PEP, the evaluation should include the observation of clinical signs of rabies (e.g., excessive salivation), the condition of the attack (provoked or not) and whether the dog is observable (20). Our results show that health professionals often rank a dog as “suspect” not only based on classic signs of rabies, but also on whether it is a “street dog,” a dog “without prior vaccination” or a dog “that cannot be observed during the 10-day period”. In particular, health professionals often reported to classify a “non-observable” dog as “suspect” instead of “healthy” and “non-observable,” which could be contributing to the observed increase in “suspect” dogs reported in Brazil (8), despite not necessarily changing the appropriate PEP to be administrated. Additionally, dog's vaccination status is not a criterion to report or used to select PEP according to the Ministry of Health guidelines. The role of street dogs in PEP use requires future research in Brazil, which has an estimated population of 25 million abandoned dogs and cats (22). In the United States, 40% of PEP use was considered inappropriate and mainly related to exposures of domestic animals not available for observations (23, 24). Implementing national strategies to reduce dog bites from street dogs could therefore be a key component in reducing unnecessary use of PEP.

Our surveys highlight the difficulty for health professionals to distinguish between a “rabid” and a “suspect” dog. In fact, most professionals used the same criteria to classify both types of dogs, 28% reported not knowing how to differentiate them, and 7% declared that they were indistinguishable. Only the observation period followed by laboratory testing upon death can definitively discriminate between both cases, allowing PEP to be discontinued if the dog is not confirmed rabid. However, only 10% of health professionals stated that laboratory testing could differentiate these dogs, highlighting the need for better training. In fact, despite a relatively high degree of confidence in assessing the dog's health condition, this misclassification could lead to excessive PEP use (e.g., RIG) in patients bitten by dogs wrongly classified as rabid. Most professionals stated that the bitten patient or the dog owner are responsible for the 10-day observation period and should report the outcome. However, patients might not be able to observe the dog or will not return to the health center (25), with 39% of professionals expressing that the patient not returning to the health center is the main obstacle to successful completion of PEP. Although dog-observation by health and veterinary services is likely to not be feasible in all cases given the number of bites registered in Brazil (more than 500.000 bite-injury patients/year), other strategies such as phone communication could be encouraged to reliably complete the dog's observation period.

Since rabies is a zoonotic disease, overcoming rabies in humans requires the use of a One Health approach including the collaboration of human and veterinary medicine (26). Our questionnaires showed that most health professionals do not consult veterinarians to assist in assessing a dog's health condition. This could be due to their confidence in reliably classifying a dog or trusting the patient's recommendation. However, almost half of dog-bite patients in Brazil do not receive the appropriate PEP including over-use (8), similar to the United States where physicians apply PEP even in low-risk exposures if the 10-day observation period cannot be assured (23). Thus, improving PEP administration by a more appropriate dog evaluation could require increasing health professional awareness of the need of a more informed opinion including veterinary help. Improving communication could benefit from including a veterinarian in the city or state's surveillance team, as expressed by health professionals, which could also contribute to the largely overlooked tracking of secondary bites of potentially rabid dogs. Specific interactions during complex cases could be promoted using mobile apps connecting professionals from these two sectors as developed for endemic rabies in other regions and for leishmaniasis in Brazil (27–29).

The high rate of PEP administrated not following the Brazilian Ministry of Health guidelines can be partly due to poor knowledge of health professionals on PEP (8). Our study supports this hypothesis, since poor knowledge on PEP administration was significantly correlated with a lack of training on PEP guidelines and low incidence of bites, similar to poor PEP administration in areas of low rabies incidence or low awareness in the United States (23). In our study, PEP KS did not increase with the number of other professionals present to support the PEP decision, which could be partially explained by a lack of communication among professionals, with 82% reporting not asking for help when completing the form. Moreover, PEP knowledge was not higher in more experienced professionals, in contrast to more experienced workers having more awareness of practices to control rabies in India (12). Most health professionals (97%) in our study were unable to correctly classify wounds as “severe” or “light” according to Ministry of Health guidelines, which also strongly influences PEP outcome in Brazil and other countries (27, 30). Therefore, our study suggests that more frequent training should help increase PEP knowledge and reduce its misuse. Future studies could also test other individual factors that can influence PEP knowledge not included in this study such as the professional's socio-economic background, risk perception, specific profession, age and gender.

Besides providing the appropriate PEP to people at risk, adequate surveillance is also key in reducing rabies burden (31). Reducing data entry errors or inconsistency and ensuring form completion are key to using SINAN in local and national surveillance for rabies (8). However, many SINAN records include major errors (e.g., rabies false positives) or missing data (8, 30). Errors in the SINAN data could arise from the relatively limited amount of time (<20 min for most professionals) to complete the form, the large number of fields (e.g., 60), errors when digiting paper forms, and a lack of feedback about errors (e.g., false positives) detected. In fact, 75% of participants did not receive feedback on the forms. Lack of feedback was reflected in less than half of professionals knowing if dog or cat rabies cases were reported in their state or municipality in recent years. Thus, we suggest that improving form completion and accuracy could be achieved by online training that highlights the utility and use of these data. For example, a scenario-based online module was successfully applied to increase knowledge of rabies and PEP in the USA (32), while a user-friendly mobile App guiding rabies prophylaxis among health-care professional was successfully implemented in India (33). Likewise, a mobile-phone-based health tool (mHealth) facilitated large-scale data collection on rabies in Tanzania, triggering automated text messages (SMS) to alert patients of vaccination schedules (34).

Overall, this study identified several knowledge gaps of health professionals assessing bite patients in Brazil, from evaluating the dog's health condition to selecting the appropriate PEP regimen. This lack of knowledge could contribute to the observed misuse of PEP over the past decade (8). Our results highlight that in Brazil, a decrease in dog and human rabies does not necessarily generate a proportional reduction in PEP demand as previously suggested in Latin America (35), unless appropriate training on dog's health condition and reduction of bites caused by “non-observable dogs” is achieved. Improving communication between public health professionals and veterinarians using One Health approaches could improve PEP administration and can potentially be achieved by applying an approach of Integrated Bite Case Management, which has previously shown potential to reduce PEP use by 40–60% through more accurate identification of dogs posing a risk for rabies (7, 36).

## Data Availability Statement

The raw data supporting the conclusions of this article will be made available by the authors, without undue reservation.

## Ethics Statement

The studies involving human participants were reviewed and approved by National Platform Brazil of the National Committee of Health of Brazil (project number: 3.029.348). The patients/participants provided their written informed consent to participate in this study.

## Author Contributions

JAB, KH, and JM conceived and designed the study. RMDS collected and analyzed the data. JAB analyzed the data. RMDS, JAB, and KH drafted the article and edited the article. AASC, CSH, and FSM supported data collection. All authors read, commented, and approved the final manuscript.

## Funding

RMDS, JM, JAB, and KH are funded by a Wellcome Trust grant (207569/Z/17/Z) awarded to KH. The funders had no role in study design, data collection and analysis, decision to publish, or preparation of the manuscript.

## Conflict of Interest

The authors declare that the research was conducted in the absence of any commercial or financial relationships that could be construed as a potential conflict of interest.

## Publisher's Note

All claims expressed in this article are solely those of the authors and do not necessarily represent those of their affiliated organizations, or those of the publisher, the editors and the reviewers. Any product that may be evaluated in this article, or claim that may be made by its manufacturer, is not guaranteed or endorsed by the publisher.
