# Supplementary material for: Factors Limiting the Appropriate Use of Rabies Post-exposure Prophylaxis by Health Professionals in Brazil
Source: Front Vet Sci. 2022 May 6;9:846994. doi: 10.3389/fvets.2022.846994 (PMC9120864; doi:10.3389/fvets.2022.846994)
Supplement: Supplementary file 1 [file Table_1.docx]

Supplementary Material

**Supplementary Material Table S1: Survey’s questionnaire**

Health Unit / Health Region / Municipality

**SECTION 1: DOG CONDITION**

| 1 – What criteria you use to classify a dog as ‘Suspect’ when filling the form?  Mentioned: Aggression, biting objects and animals, anorexia, sialorrhea, behavior change, agitation, bark change, seizures, ataxy, bite on the neck/head, no prior vaccination, dies after the bite, paralysis, hydrophobia, neurological signs, photophobia, disappeared dog, street dog, diagnosis test, non-observable dog, doesn’t know, ownerless dog, unknown dog, other |
| --- |
| 2 – What criteria you use to classify a dog as ‘Rabid’ when filling the form?  Mentioned: Aggression, biting objects and animals, anorexia, sialorrhea, behavior change, agitation, bark change, seizures, ataxy, bite on the neck/head, no prior vaccination, dies after the bite, paralysis, hydrophobia, neurological signs, photophobia, disappeared dog, street dog, diagnosis test, non-observable dog, doesn’t know, ownerless dog, unknown dog, other |
| 3 – How do you differentiate a ‘Suspect’ dog from a ‘Rabid’ dog?  Mentioned: Presence of specific signs; Clinical signs; Different symptoms; Rabid has rabies signs and suspect by other factors; Diagnosis test; No difference; Patient’s assessment; Other |
| 4 - What is the criteria to classify a dog as ‘Observable’?  Mentioned: Has owner and lives in a fix location (no access to street); Has owner, but lives in the street; Has no owner, but is a community dog and can be found; Dog isn't dead; Dog in fixed location; Patient says if he is going to observe the dog; Doesn’t know; Other. |
| 5- If a dog presenting rabies signs disappear after a bite/scratch, how do you classify the dog?  Rabid; Suspect; Disappeared; Dead; Doesn’t know |
| 6 – Do you consult a veterinary?  No; Yes, to determine the general condition of the dog before the prescription of PEP; Yes, to help in the 10-day period observation; Doesn’t Know, Other |
| 7 – Who’s in charge of following the dog during the 10-day period observation?  Mentioned: Nurse/Doctor; Patient; Private veterinary; City’s veterinary; Surveillance team; Zoonosis center; The animal owner; Don’t know; Other |
| 8 – How do you receive the observation outcome?  Mentioned: You call the patient; Health center calls the patient; The patient returns to the health center; You go to the patient; Surveillance team refers the result; Health agent does active search; City’s veterinary is responsible; Doesn’t receive the outcome; Other |
| 9 – What happens if you don’t receive the observation outcome?  Mentioned: You call the patient; Health center calls the patient; You go to the patient’s house; Close the case; Get in contact with the zoonosis center; Surveillance team receive the outcome; Health agent does active search; Always receive the outcome; Nothing; Other |
| 10 – Do you try to find other people bitten by the same animal?  Yes; No; It depends |
| 11 – Which of these wounds you consider as a ‘severe’ wound?  Licking on mucosa; Licking on hands; Superficial wound on the feet; Superficial wound on the glutes; Punctiform injuries without bleed; Punctiform injuries with bleeding; Multiple lights wounds on the back; Doesn’t know |
| 12 – Do you usually believe in the patient’s report on the dog?  Yes; No |
| 13 – How often you know how to classify the dog status?  Never; Occasionally; Often; Always |
| **SECTION 2: PEP ADMINISTRATION**  14 – What is the main criteria to decide to administrate the PEP?  Mentioned: Dog’s general condition; Severity of the wound; Availability of the PEP; Rabies cases in the area; Cost for the health unit; No specific factor; Animal species responsible for the bite; All alternatives; Doesn’t know; Other |
| 15 – How many doses you must administer to a patient with severe wounds from a disappeared/dead dog?  0; 1; 2; 3; 4; 5; Doesn’t know |
| 16 – If a patient arrives presenting a scratch on his arm by a disappeared dog, which treatment would you recommend?  No treatment; Vaccine; Vaccine and serum; Doesn’t know |
| 17 - If a patient arrives presenting two bites on his hand by a healthy dog that has owner, which treatment would you recommend?  Observation only; Vaccine and observation; Vaccine without observation; Vaccine and serum with observation; Vaccine and serum without observation; No treatment; Doesn’t know  *If observation: what do you do if the dog disappears or dies within the 10 days?*  Stop treatment  Continue same treatment  Changes treatment, to: |
| 18 - If a patient arrives presenting a bite on his neck by a street dog that wasn’t provoked and has no owner, which treatment would you recommend?  Observation only; Vaccine and observation; Vaccine without observation; Vaccine and serum with observation; Vaccine and serum without observation; No treatment; Doesn’t know  *If observation: what do you do if the dog disappears or dies within the 10 days?*  Stop treatment  Continue same treatment  Changes treatment, to: |
| 19 – What’s the main obstacle to correctly administer PEP?  Mentioned: Vaccine not available; Difficulty in evaluating the dog’s condition; Data given by the patient isn’t reliable; Patient doesn’t return to finish PEP; Lack of training; Vaccine centralization discourages the patient; Disagreement between the team; No obstacle; Doesn’t know; Other |
| 20 - Do patients demand for a specific PEP regimen, regardless of your assessment? If yes, how do you respond?  Yes; No. If yes, usually ignores the indication of the patient? Yes; No |
| 21 – Does your colleagues ask for you to apply a specific PEP regimen in some cases, regardless of your assessment? If yes, how do you respond?  Yes; No. If yes, usually ignores the indication of the patient? Yes; No |
| 22 – How much confidence you have in applying PEP?  None; Low; Medium; High |
| 23 – How often you know which PEP to apply?  Always; Often; Sometimes; Never |
| 24 – If you have any doubt on the correct administration of PEP, what do you do?  Mentioned: Ask a colleague; Search in the protocol; Ask a superior; Call a professional who has the knowledge; Doesn’t know; Other  25 – If you believe that a patient was bitten by a rabid dog, what do you do?  Mentioned: Call a veterinary for help; Ask a veterinary to follow the animal and collect samples; To inform the city’s zoonosis center; Follow the usual procedure and doesn’t warn any authority; Warns his health superior; Calls the health secretary; Forward to the referral hospital; Doesn’t know what to do; Other |
| **SECTION 3: SINAN FORM**  26 – Do you understand all the record fields on the SINAN form?  Yes; No |
| 27 – Do you usually fill all the record fields on the SINAN form?  Yes; No, the form is too long; No, it’s hard to understand; No, it’s not necessary; No, the information is not used; No, some of the information is filled until the end of the treatment; No, other |
| 28 – How long do you take to fill the SINAN form?  0-10 min; 11-20 min; 21-30 min; 30 min-1h; >1h |
| 29 – Do you ask regularly to colleagues how to fill the SINAN form?  Yes; No |
| 30 – How would you prefer to fill the SINAN form?  Paper sheet; Computer; Mobile app |
| 31 – When do you fill the SINAN form?  When the patient is assessed; At the end of my shift; Next day; When the patient returns |
| 32 – What is the SINAN form used for? Mention two applications: |
| 33 – Do you think that the SINAN form is used to improve PEP administration?  Yes; No |
| 34 – For how many years have you fill out the SINAN form? |
| 35 – How many people that compose your team can fill the SINAN form? |
| 36 – Do you receive any feedback on the SINAN reports?  Yes; No |
| 37 – What have you been trained for?  Filling the SINAN form; When/how to apply PEP; No training |
| 38 – How to improve interactions with veterinarians?  Mentioned: Having a veterinary of the city or in the surveillance team; Having a private veterinary contact; Having meeting with the veterinary from the city or in the surveillance team; To have training along with veterinarians; Doesn’t know; Other |
| **SECTION 4: DOG RABIES SITUATION IN THE AREA**  39 – Do you receive feedback from your superiors regarding the rabies situation in your city/state?  No; Yes, monthly; Yes, annually; Yes, irregular and informal |
| 40 - Have there been any cases of rabies in dogs or cats in the last 2 years in your state?  Yes; No; Doesn’t know |
| 41 - Have there been any cases of rabies in dogs or cats in the last 5 years in your municipality?  Yes; No; Doesn’t know |
| 42 - Have there been any cases of rabies in wild animals in the last 2 years in your municipality?  Yes; No; Doesn’t know |
| 43 - Have there been any cases of rabies in wild animals in the last 2 years in your state?  Yes; No; Doesn’t know |
| 44 – Is rabies vaccine always available in you unit?  Yes; No; Doesn’t know |
| 45 – Is rabies serum always available in your unit?  Yes; No; Doesn’t know |
